# Women in Bioorganic Chemistry

**DOI:** 10.3390/molecules27134290

**Published:** 2022-07-04

**Authors:** Francesca Cardona, Camilla Parmeggiani, Camilla Matassini

**Affiliations:** 1 Dipartimento di Chimica “Ugo Schiff” (DICUS), Università di Firenze, via della Lastruccia 3-13, 50019 Sesto Fiorentino, Italy; camilla.parmeggiani@unifi.it (C.P.); camilla.matassini@unifi.it (C.M.); 2 European Laboratory for Non Linear Spectroscopy (LENS), via Nello Carrara 1, 50019 Sesto Fiorentino, Italy

We are very happy to present this Special Issue, for which we acted as guest editors, and which includes scientific contributions from laboratories headed by women active in the field of bioorganic chemistry.

We made the decision to undertake this project since we deem that there are still gender biases that put women at a slight disadvantage when disseminating their research, preventing the science community from benefiting from a wider diversity of voices. The issues related to the gender scissor and the leaky pipeline that can be observed with career advancement in the academy, especially in the field of STEM disciplines, deserve our attention and the efforts of all of the scientific community to mitigate the gender gap. In order to embrace gender equality, recognize the career progression of women, and to celebrate the achievements of women in the field of bioorganic chemistry, we present in this Special Issue contributions both from highly renewed woman scientists and young woman researchers who are undertaking their early-stage careers.

This Special Issue includes fifteen manuscripts, among which eleven high-quality research articles and four comprehensive review articles in the area of bioorganic chemistry, published from mid-2020 to early 2022.

The scope of the Special Issue covers a wide range of topics at the organic chemistry-biology interface, including the synthesis and derivatization of natural compounds and their analogues, and the investigation of their biological activities in the human health field (for instance as antitumoral, antioxidants and antimicrobial agents) as well as their possible application in the crop protection field as agrochemicals. An example of nanoparticle-based biomaterial is also included. The techniques employed, besides organic synthesis, are in silico studies (docking procedures and molecular modeling), FT-IR spectroscopy, laser diffraction, PET, fluorescence, STD-NMR studies, enzymatic evaluation, experiments on cell lines, and in vivo studies on mice.

Cardona, Matassini and co-workers, from Sesto Fiorentino, Italy, reviewed the properties of carbohydrate-based natural compounds and other sugar mimics as trehalase inhibitors, in view of their potential use as non-toxic and therefore greener and safer pesticides [1].

Within the same field of agrochemicals, Dell’Oste, Spyrakis, Prandi and co-workers from Torino, Italy, described that strigolactones (SG), a class of sesquiterpenoid plant hormones, play a key role in the plants’ response to biotic and abiotic stress. In addition, the authors highlighted the possibility that in the next future these compounds might have an application also in human health, and in particular in the control pathways related to apoptosis and inflammation (and therefore as anticancer and/or antimicrobial agents) [2]. Terpenes have a number of other different biological applications, as reported by the authors of this Special Issue. Zhan and co-workers, from Guangzhou, China, showed that the natural compound betulinic acid (BA), a pentacyclic triterpene widely distributed in nature, behave as a non-competitive inhibitor of α-glucosidase, showing a synergistic effect with acarbose, which is known for its use for alleviation of post-prandial hyperglycemia. The authors also performed molecular docking and molecular dynamics simulation and some preliminary in vivo experiments on mice [3]. Mazzon and co-workers, from Messina, Italy, reported on the numerous studies supporting the great properties of cannabidiol (CBD), a terpenophenol natural compound, for the management of neurological disorders (such as epilepsy, Alzheimer, multiple sclerosis and Parkinson), due to is antioxidant, anti-inflammatory, antidepressant, anxiolytic, anticonvulsant and antipsychotic properties. The biochemical and molecular mechanisms underlying the effects of CBD show that a multi-target mechanism of action takes place [4]. Nesterkina and co-workers from Odessa, Ukraina studied, with the aid of different techniques (FT-IR, laser diffraction, fluorescent measurements), the impact of terpenoids-based hydrazones on the molecular organization of lipid matrices using model liposomes based on lecithin or cardiolipin phospholipids, as well as lipids isolated from rat strata cornea [5].

Triterpenes are biosynthetic precursors of steroids, which are an important class of both natural and synthetic products. Volkova and co-workers from Moscow, Russia, described the synthesis of d-annulated pentacyclic steroids based on a regioselective interrupted Nazarov cyclization with trapping chloride ion, and evaluated the antiproliferative activity of the synthesized compounds against two breast cancer cell lines [6]. 

The interest in the design and synthesis of novel anticancer therapeutics is also present in the manuscript by Beloglazina and co-workers from Moscow, Russia, who reported the synthesis of a series of S-, O- and Se- containing dispirooxoindoles through 1,3-dipolar cycloaddition of azomethine ylides, assayed their cytotoxicity against different tumor cell lines and performed an in silico study to rationalize the results [7]. The group of Simone and co-workers from Callaghan, Australia, reported the synthesis, glycosidase inhibition and anticancer properties of highly chlorinated benzamide analogues bearing a boron-pinacolate ester group, with the perspective to use them in boron neutron capture therapy (BNCT) [8].

Sattin and co-workers from Milano, Italy, described, through virtual screening accompanied by STD-NMR studies, a structure-based approach to find new chemotypes able to target (p)ppGpp (guanosine tetra-or penta-phosphate) signaling, in view of overcoming antimicrobial resistance [9]. The issue of antimicrobial resistance was also addressed by the group of Grosdemange-Billiard and co-workers from Strasbourg, France, who synthesized fluorinated analogues of the natural compound fosmidomycin and tested them as *E. coli* 1-deoxy-d-xylulose 5-phosphate reductoisomerase (DXR) inhibitors as well as antimicrobial agents against *E. coli* on Petri dishes [10]. Pathogenic *E. coli* infection and food/water contamination by this pathogen was also the object of the article by Wu and co-workers from Nanchang, China, who designed and synthesized a β-galactosidase-activatable fluorescent probe (BOD-Gal) for the detection of this pathogen [11].

The process of microbial attack on dental enamel and the potential approaches for dental remineralization were described by Brimble and co-workers from Auckland, New Zealand, who highlighted the importance of the amelogenin protein and the efforts made by the researchers in the identification of the key structural motifs of this protein that enable dental remineralization, as well as the rational design of synthetic polypeptides for this aim [12].

Integrin α_4_β_1_ belongs to the leukocyte integrin family and represents a target of relevant therapeutic interest due its role in mediating inflammation, autoimmune pathologies and cancer-related diseases. With the aim of discovering new compounds potentially able to recognize integrin α_4_β_1_, Battistini and co-workers from Parma, Italy synthesized, through solid phase procedures followed by in-solution cyclization steps, seven new cyclic peptidomimetics bearing a 4-aminoproline core scaffold, and evaluated them in cell adhesion assays on Jurvat cells [13].

In the field of bionanomaterials, the Special Issue shows an example by Bodlenner from Strasbourg, France, and Matassini from Sesto Fiorentino, Italy, who reported the synthesis and biological evaluation as Jack Bean α-mannosidase inhibitors of hybrid multivalent glyco gold nanoparticles decorated with deoxynojirimycin inhitopes, among the best known glycomimetics in the field of glycosidases inhibition. The authors found a strong enhancement of the inhibitory activity consequent to the multivalent presentation of the inhitope [14].

Lastly, chirality is one of the most crucial aspects of nature and is of paramount importance in the area of bioorganic chemistry, and axial chirality represents an intriguing aspect of chirality itself. Viglianisi and co-workers from Sesto Fiorentino, Italy, developed an efficient chemical resolution of racemic aza[4]helicenes, interesting building blocks for the production of materials with chiroptical properties, using enantiopure camphanic acids as resolving agents [15].

We want to finish this Editorial by thanking again all of the authors who come from three different continents, namely Europe, Asia and Oceania, for having illustrated so well the importance of bioorganic chemistry in their contributions to this Special Issue.

A list of short biographical sketches of the authors, together with the description of the obstacles/challenges encountered during their career, or suggestions to a young woman keen to become a successful scientist in the field of bioorganic chemistry, follows in the Reference section.
